# Cardio-Obstetrícia: Uma Subespecialidade Essencial em Ascensão

**DOI:** 10.36660/abc.20230433

**Published:** 2024-03-21

**Authors:** Walkiria Samuel Avila, Alexandre Jorge Gomes de Lucena

**Affiliations:** 1 Hospital das Clínicas da Faculdade de Medicina da Universidade de São Paulo Instituto do Coração São Paulo SP Brasil Instituto do Coração do Hospital das Clínicas da Faculdade de Medicina da Universidade de São Paulo, São Paulo, SP – Brasil; 2 Hospital Agamenon Magalhães Recife PE Brasil Hospital Agamenon Magalhães, Recife, PE – Brasil

**Keywords:** Cardiopatias, Gravidez, Tutoria

Nas últimas décadas, a doença cardiovascular tornou-se a principal causa de morte relacionada à gravidez. Esse fato deve-se à crescente prevalência de fatores de riscos para doença cardiovascular em mulheres jovens, ao planejamento da gestação em faixa etária mais tardia da vida reprodutiva, e ao progressivo número de cardiopatias congênitas no adulto.^1 , 2^

No Brasil, estima-se que 4% das gestações ocorram em mulheres com cardiopatias, e essas representam a principal causa não obstétrica de morte materna. Acrescenta-se que as causas evitáveis ou provavelmente evitáveis dessas mortes alcançam índices de até 80%, e que dois terços dos óbitos ocorrem ao longo de 12 meses após o parto.^3^

Ademais, estudos recentes demonstram que complicações específicas da gravidez como a doença hipertensiva, diabetes gestacional, parto prematuro e restrição de crescimento intrauterino, estão associadas a maior risco de hipertensão arterial, doença cardíaca isquêmica, e insuficiência cardíaca ao longo da vida. Isso representa um importante impacto na carga global de mortalidade no sexo feminino.^4^

Diante desse cenário, é de se supor que a carência de conhecimentos específicos sobre os riscos imediatos e tardios impostos pela gravidez prejudica o aprimoramento na prevenção da mortalidade decorrente das doenças cardiovasculares entre as mulheres.^5^ Nesse sentido, a proposição em constituir a Cardio-obstetrícia, como uma subespecialidade da Cardiologia, está em franca expansão em todo o mundo.^6 – 8^

A parceria de obstetras e ginecologistas com outros médicos especialistas nas condutas durante a gestação e após o parto, na avaliação dos riscos antes da concepção, e na atenção primária à saúde da mulher com histórico de gravidez complicada, é a "pedra angular" da Cardio-obstetrícia. Essa interdisciplinaridade norteia decisões de conduta apoiadas na discussão com os especialistas qualificados e treinados e valoriza a Bioética para a integração ética, moral e legal acerca das responsabilidades inerentes aos especialistas envolvidos.^9 , 10^ ( Figura 1 )

**Figura 1:**
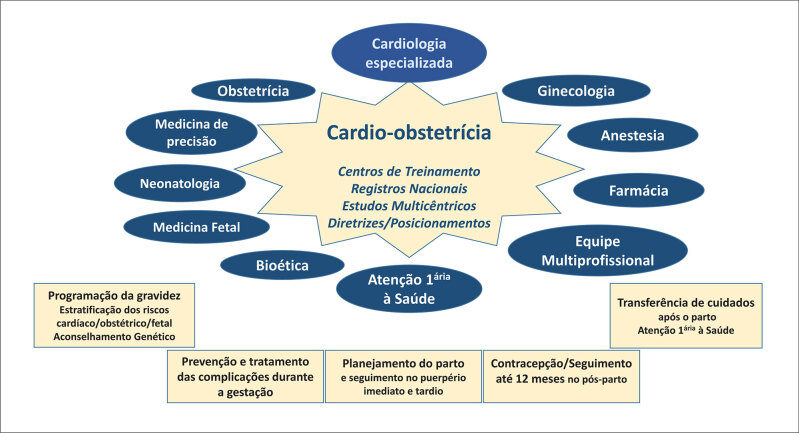
Atuação da Cardio-obstetrícia na assistência, ensino e pesquisa relativo à gravidez em pacientes poradoras de cardiopatias.

A Cardio-obstetrícia é uma especialidade em evolução, com Posicionamentos e Diretrizes próprios para os diversos padrões de atendimento, ampliando os horizontes e solidificando os alicerces da interdisciplinaridade de acordo com as demandas crescentes da população. Além do mais, os desafios éticos e o escasso investimento em ensaios clínicos na gestação impõem a elaboração de registros nacionais e estudos multicêntricos na busca de evidências para a prática clínica.

Na verdade, os programas de residência em Cardiologia não incluem a Cardio-obstetrícia no domínio de formação e tampouco a admitem como uma área importante para uma promissora carreira.^11^ Assim, a falta de treinamento adicional não permite o avanço dessa subespecialidade que permanece "nas mãos de poucas mãos.^12^

Nesse sentido, há de se destacar que profissionais integrantes do Instituto do Coração do Hospital das Clínicas da Faculdade de Medicina da Universidade de São Paulo (InCor-HCFMUSP) construíram um portfólio de expertise plenamente reconhecido. Hoje, ele corresponde ao Núcleo de Ensino e Pesquisa em Cardiopatia e Gravidez e Aconselhamento Reprodutivo-InCor, que tem cumprido nas quatro últimas décadas^13^ o compromisso de ser um modelo em capacitação além de despertar a escolha da Cardio-obstetrícia como uma carreira médica.

Ainda assim, o Programa de Residência Médica em Cardiologia de Adultos do InCor – 2022 que tem o intuito de se adequar à realidade do mercado de trabalho nacional não incluiu a Cardio-obstetrícia na grade curricular de prevenção cardiovascular e promoção da saúde no adulto.^14^ Certamente, as comissões responsáveis pelos ajustes nos programas de residência médica deverão se alinhar às perspectivas mundiais no reconhecimento da Cardio-obstetrícia como subespecialidade singular e essencial na formação do cardiologista.

## References

[1] Brasil. Ministério da Saúde (2020). Secretaria de Vigilância em Saúde. Boletim Epidemiológico.

[2] Collier AY, Molina RL (2019). Maternal Mortality in the United States: Updates on Trends, Causes, and Solutions. Neoreviews.

[3] Campanharo FF, Cecatti JG, Haddad SM, Parpinelli MA, Born D, Costa ML (2015). The Impact of Cardiac Diseases During Pregnancy on Severe Maternal Morbidity and Mortality in Brazil. PLoS One.

[4] Parikh NI, Gonzalez JM, Anderson CAM, Judd SE, Rexrode KM, Hlatky MA (2021). Adverse Pregnancy Outcomes and Cardiovascular Disease Risk: Unique Opportunities for Cardiovascular Disease Prevention in Women: A Scientific Statement from the American Heart Association. Circulation.

[5] Shapero KS, Desai NR, Elder RW, Lipkind HS, Chou JC, Spatz ES (2020). Cardio-Obstetrics: Recognizing and Managing Cardiovascular Complications of Pregnancy. Cleve Clin J Med.

[6] Sharma G, Zakaria S, Michos ED, Bhatt AB, Lundberg GP, Florio KL (2020). Improving Cardiovascular Workforce Competencies in Cardio-Obstetrics: Current Challenges and Future Directions. J Am Heart Assoc.

[7] Thakkar A, Hailu T, Blumenthal RS, Martin SS, Harrington CM, Yeh DD (2022). Cardio-Obstetrics: The Next Frontier in Cardiovascular Disease Prevention. Curr Atheroscler Rep.

[8] Graves CR, Woldemichael RM, Davis SF (2023). Cardio-Obstetrics: Moving Beyond Programming to Action. J Am Heart Assoc.

[9] Grewal J, Windram J, Silversides C (2021). Cardio-Obstetrics: Past, Present and Future. Can J Cardiol.

[10] Grodzinsky A, Florio K, Spertus JA, Daming T, Lee J, Rader V (2019). Importance of the Cardio-Obstetrics Team. Curr Treat Options Cardiovasc Med.

[11] Bello NA, Agrawal A, Davis MB, Harrington CM, Lindley KJ, Minissian MB (2022). Need for Better and Broader Training in Cardio-Obstetrics: A National Survey of Cardiologists, Cardiovascular Team Members, and Cardiology Fellows in Training. J Am Heart Assoc.

[12] Minhas AS, Goldstein SA, Vaught AJ, Lewey J, Ward C, Schulman SP (2022). Instituting a Curriculum for Cardio-Obstetrics Subspecialty Fellowship Training. Methodist Debakey Cardiovasc J.

[13] Avila WS, Rossi EG, Ramires JA, Grinberg M, Bortolotto MR, Zugaib M (2003). Pregnancy in Patients with Heart Disease: Experience with 1,000 Cases. Clin Cardiol.

[14] Lottenberg MP, Bichuette LD, Bortolotto LA, Gowdak LHW, Darrieux FCDC, Binotto MA (2022). Incor Residency Program in Adult Cardiology in 2022: 40 Years Preparing Cardiologists for the Demands in Brazil. Arq Bras Cardiol.

