# Supplementary material for: Cardio-Obstetrícia: Uma Subespecialidade Essencial em Ascensão
Source: Arq Bras Cardiol. 2024 Mar 21;121(2):e20230433. [Article in Portuguese] doi: 10.36660/abc.20230433 (PMC12092028; doi:10.36660/abc.20230433)
Supplement: Translation

# Translation: Cardio-Obstetrics: A Critical, Growing Subspecialty

**Authors:** Walkiria Samuel Avila, Alexandre Jorge Gomes de Lucena

**Affiliations:** 1 São Paulo SP Brazil Instituto do Coração do Hospital das Clínicas da Faculdade de Medicina da Universidade de São Paulo, São Paulo, SP – Brazil; 2 Recife PE Brazil Hospital Agamenon Magalhães, Recife, PE – Brazil

**Keywords:** Heart Diseases, Pregnancy, Mentoring

In the last decades, cardiovascular diseases have become the leading cause of pregnancy-related deaths. This is due to the increasing prevalence of risk factors for cardiovascular disease in young women, late pregnancy planning, and the increasing number of cases of adult congenital heart disease.^1 , 2^

In Brazil, it is estimated that 4% of pregnancies occur in women with heart disease, which represents the main non-obstetric cause of maternal death. In addition, the preventable and probably preventable causes of deaths reach up to 80%, and two thirds of deaths occur within 12 months after delivery.^3^

Also, recent studies have shown that specific complications of pregnancy such as hypertensive disease, gestational diabetes, premature delivery, and intrauterine growth restriction, are associated with a higher risk of arterial hypertension, ischemic heart disease, and heart failure over lifetime. This represents an important impact on global mortality burden in women.^4^

In this scenario, one may assume that advances in prevention of cardiovascular mortality in women are difficult because of the lack of knowledge about pregnancy-related short- and long-term risks.^5^In this regard, the interest in developing Cardio-obstetrics as a subspecialty of cardiology has grown worldwide.^6 – 8^

The collaboration of obstetricians, gynecologists and other medical specialists during pregnancy and postpartum care, in the assessment of risks before and after conception, and in primary care of women with a history of complicated pregnancy is the cornerstone of Cardio-obstetrics. This interdisciplinarity provides guidance based on discussions with qualified and trained experts, as well as on bioethics to integrate ethics, moral, and legal issues regarding the responsibilities of each specialist involved.^9 , 10^( Figure 1 )

**Figure 1:** The role of cardio-obstetrics in assistance, education and research related to pregnancy in cardiovascular patients.

Cardio-obstetrics is a changing subspecialty, with its own positioning and guidelines for different patterns of care, expanding horizons and laying the foundation for interdisciplinarity according to the increasing demand from the population. Besides, due to ethical challenges and poor investment in clinical trials with pregnant women, there is a need for national registries and multicentric studies to search for evidence for clinical practice.

In fact, Cardio-obstetrics is not included in the formation domain of Cardiology fellowship programs and is even not regarded as an important field for a promising career.^11^Thus, the lack of additional training limits the advance of this subspecialty, which remains in the hands of few people.^12^

It is crucial to highlight that members of the Heart Institute (InCor) of the Sao Paulo Medical School's General Hospital, University of Sao Paulo, have built a renowned expertise portfolio. Today, it corresponds to the *Núcleo de Ensino e Pesquisa em Cardiopatia e Gravidez e Aconselhamento Reprodutivo-InCor* (Center for education and research in heart disease and pregnancy and reproduction counseling-InCor). For the last four decades,^13^this center has made the commitment to be a model of capacitation and foster the choice for Cardio-obstetrics as a medical career.

Nevertheless, the InCor Adult Cardiology Fellowship Program (2022), which claims to be aimed at adapting to the Brazilian national labor, has not included "Cardio-obstetrics" in the curricular track of cardiovascular prevention and health promotion in adults.^14^Certainly, the committees responsible for the adjustments in the medical fellowship programs should align with the world's perspective on Cardio-obstetrics as a unique and essential subspecialty in Cardiology Education.
